# Comparison of plasma clearance of [^51^Cr]CrEDTA based on three, two and single samples to measure the glomerular filtration rate in patients with solid tumors: a prospective cross-sectional analysis

**DOI:** 10.1016/j.clinsp.2024.100427

**Published:** 2024-06-29

**Authors:** Anne C. Gomes, George B. Coura Filho, Luiz A. Gil Junior, Renato A. Caires, Emmanuel A. Burdmann, Carlos A. Buchpiguel, Veronica T. Costa e Silva, Marcelo T. Sapienza

**Affiliations:** a Divisão de Medicina Nuclear, Hospital das Clínicas, Faculdade de Medicina, Universidade de São Paulo (HCFMUSP), São Paulo, SP, Brazil; b Laboratório de Investigação Médica (LIM 66), Faculdade de Medicina, Universidade de São Paulo (FMUSP), São Paulo, SP, Brazil; c Serviço de Nefrologia, Instituto do Câncer do Estado de São Paulo, Faculdade de Medicina, Universidade de São Paulo (FMUSP), São Paulo, SP, Brazil; d Laboratório de Investigação Médica (LIM 12), Faculdade de Medicina, Universidade de São Paulo (FMUSP), São Paulo, SP, Brazil; e Laboratório de Investigação Médica (LIM 43), Faculdade de Medicina, Universidade de São Paulo (FMUSP), São Paulo, SP, Brazil; f Laboratório de Investigação Médica (LIM 16), Faculdade de Medicina, Universidade de São Paulo (FMUSP), São Paulo, SP, Brazil

**Keywords:** Glomerular filtration rate, Radionuclides, Edetic acid, Medical oncology

## Abstract

**Objectives:**

[^51^Cr]CrEDTA is used to measure the Glomerular Filtration Rate (GFR) in different clinical conditions. However, there is no consensus on the ideal number of blood samples to be taken and at what time points to measure its clearance. This study aimed to compare Slope Intercept (SI) and Single-Sample (SS) methods for measuring GFR in patients with solid tumors, stratified by age, GFR, and Body Mass Index (BMI).

**Methods:**

1,174 patients with cancer were enrolled in this prospective study. GFR was calculated by the SI method using blood samples drawn 2-, 4-, and 6-hours after [^51^Cr]CrEDTA injection (246-GFR). GFR was also measured using the SI method with samples at 2 and 4 hours (24-GFR) and at 4 and 6 hours (46-GFR), and SS methods according to Groth (4Gr-GFR) and Fleming (4Fl-GFR). Statistical analysis was performed to assess the accuracy, precision, and bias of the methods.

**Results:**

Mean 246-GFR was 79.2 ± 21.9 mL/min/1.73 m^2^. ANOVA indicated a significant difference between 4Gr-GFR and the reference 246-GFR. Bias was lower than 5 mL/min/1.73 m^2^ for all methods, except for SS methods in subgroups BMI > 40 kg/m^2^; GFR > 105 or < 45. Precision was adequate and accuracy of 30 % was above 98% for all methods, except for SS methods in subgroup GFR < 45.

**Conclusion:**

46-GFR and 246-GFR have high agreement and may be used to evaluate kidney function in patients with solid tumors. Single-sample methods can be adopted in specific situations, for non-obese patients with expected normal GFR.

## Highlights

• EDTA clearance can be used to measure GFR in patients with solid tumors.
• Precise GFR assessment is crucial to optimize therapy and minimize side effects.
• There is a high agreement between two (46GFR) and three-time samples (246GFR).
• Single-sample tests may be adopted for non-obese patients with expected normal GFR.

## Introduction

Accurate assessment of Glomerular Filtration Rate (GFR) in patients with cancer is important to determine eligibility for specific therapies or Randomized Clinical Trials (RCT) and to adjust the dosing of chemotherapy to minimize risks of under-treatment and unnecessary systemic and kidney toxicity.^1^^,^^2^

In clinical practice, GFR is usually estimated using equations based on the Serum level of Creatinine (SCr).^3^ However, SCr is determined not only by GFR but also by diet and muscle mass. In consequence, GFR estimation using equations based on SCr (eGFRcr) may be associated with large errors in the elderly, in sarcopenic patients, or in those with rapid weight decline, scenarios often seen in cancer patients.^4^ eGFRcr also presents limitations for obese patients and for those with low GFR, also a concern in oncology, as up to 25% of cancer patients have Chronic Kidney Disease (CKD), two-thirds of them with GFR between 30 and 60 mL/min/1.73 m^2^.^2^^,^^5^^,^^6^ Cystatin C (Cys C) is recommended in the overall population for estimation of GFR when eGFRcr is thought not to be accurate.^7^ However, the serum level of Cys C may be increased in the presence of smoking, inflammation, adiposity, (hyper) thyroid function, and the use of glucocorticoids (doi:10.1038/ki.2008.638). For these reasons, direct measurement of GFR has been increasingly used during cancer treatment and advocated by recent guidelines for the prescription of platins and methotrexate, and in circumstances in which GFR estimation is expected to be inaccurate.^8^

Several techniques for directly measuring GFR based on the serum or urinary clearance of exogenous filtration markers are available in clinical practice, including the plasma clearance of Chromium-51 labeled Ethylene Diamine Tetra-acetic Acid ([^51^Cr]CrEDTA), which is almost unbiased compared to the urinary clearance of inulin, the gold standard for GFR measurement.^9^, ^10^, ^11^, ^12^ The early and late exponential components of the EDTA clearance can be evaluated by the full concentration-time curve with multiple samples (8 or more). However, it adds significant complexity to the procedure and may not even provide an accurate estimation of the early clearance component (extravascular distribution).

The most commonly used alternative is the assessment of EDTA clearance based on late samples (≥ 2 hours), applying the Slope Intercept (SI) method to calculate the slow clearance component (kidney clearance). The systematic error of not including the fast exponential component is corrected by procedures such as the one proposed by Brochner-Mortensen.^12^ Another simplification of [^51^Cr]CrEDTA GFR is the use of Single-Sample (SS) methods, including the mean sojourn time-based methodology developed by Growth^13^, ^14^, ^15^ and the method described by Fleming.^16^ The SS_GFR method, as outlined by Fleming, originates from the Jacobsson equations.^17^ It offers the benefits of decreased systematic and random errors and is presently endorsed by the British Nuclear Medicine Society as the recommended approach.^18^

SI or SS GFR are equally accurate in most patients.^19^^,^^20^ The 2018 BSNM guidelines recommended the use of SS for routine clinical measurement of GFR, except for patients with ascites, edema, or other expanded fluid space.^18^ However, a decreased accuracy of [^51^Cr]CrEDTA GFR is described for patients with pronounced loss of muscle mass or cachexia, and low GFR, especially when using SS methods,^19^^,^^21^ which could lead to less reliable results, particularly in cancer patients. Late samples are necessary when low GFR is expected (based on SCr measurements, previous GFR studies, or clinical data).^22^^,^^23^ However, there is no consensus on the optimal number of blood samples to collect and at what time points,^19^, ^24^, ^25^, ^26^ and if the protocol should be tailored according to the clinical characteristics of patients.

This study aimed to compare different SI and SS methods in GFR measurement based on the plasma clearance of [^51^Cr]CrEDTA in patients with solid tumors, stratified by age, GFR, and Body Mass Index (BMI). Results were compared to the SI method with three samples, collected at two, four, and six hours.

## Methods

Patients were enrolled in a prospective cohort (Onco-GFR Study) conducted in the studied institution, including mGFR and blood laboratory exams. A detailed description of the study protocol is provided elsewhere.^27^ Briefly, adult patients with solid tumors confirmed by histology, ECOG-PS (Eastern Cooperative Oncology Group performance status) ≤3, with no recent cancer treatment and no current evidence of risk factors for acute GFR decline, were invited to participate. The study was approved by the Institution and Brazilian Ethics Committee (CEP, number 387/14). All patients gave written informed consent. This study is reported according to the STROBE (Strengthening the Reporting of Observational Studies in Epidemiology) Statement.

### GFR measurement

GFR was measured by plasma clearance of [^51^Cr]CrEDTA at the Nuclear Medicine Center (described in detail in Supplementary Material 1: [^51^Cr]CrEDTA exam). Briefly, the reference method (246-GFR) was based on three blood samples, drawn two, four, and six hours after administration of 3.7 MBq (100 µCi) of [^51^Cr]CrEDTA. Plasma samples were counted in a well counter and GFR was calculated through the slope-intercept method described by Bröchner-Mortensen.^12^ indexed to 1.73 m^2^ of Body Surface Area (BSA).^28^ Only studies with a correlation coefficient R^2^ greater than 0.975 for all samples were included for analysis.

Results obtained using the SI method with two samples drawn at two and four hours (24-GFR) or at four and six hours after [^51^Cr]CrEDTA injection (46-GFR) were compared to the reference method. SS GFR using the four hours sample according to Growth^13^ and Fleming^16^ techniques (4Gr-GFR and 4Fl-GFR) was also compared to the reference method.

Other sampling times were evaluated and presented as supplementary material: SI GFR with two samples at two and six-hour (26-GFR); SS GFR according to Groth and Fleming methods using the two-hour sample (2Gr-GFR and 2Fl-GFR), the six-hour sample (6Gr-GFR and 6Fl-GFR), or the combination of SS with sampling times adjusted according to the expected GFR.

### Statistical analysis

Results of the different GFR estimation methods were described for the whole population and subgroups based on age (> 65 years), BMI (> 30 and > 40 kg/m²), and 246-GFR (< 45, 45‒60, 60‒90, 90‒105 and > 105 mL/min/1.73 m^2^. Analysis of variance (one-way ANOVA with repeated measures) with Dunnett´s post-test was used to compare every mean to the control (246-GFR), considering a significance level (α) of 0.05. Bias was defined as the mean difference between the 246-GFR and other methods, precision as the standard deviation of the difference between 246-GFR and other methods, and accuracy as the percentage of the results lying within 30% and 10% of the 246-GFR (respectively, Acc30% and Acc10%). Bland-Altman analysis (B&A) was performed to evaluate differences between the GFR methods, considering 246-GFR as the standard. Deming regression analysis was additionally conducted, under the assumption that the reference method (246-GFR) may also be susceptible to errors with a similar level of uncertainty as the two-sample or SS methods.

## Results

**Fig. 1:**
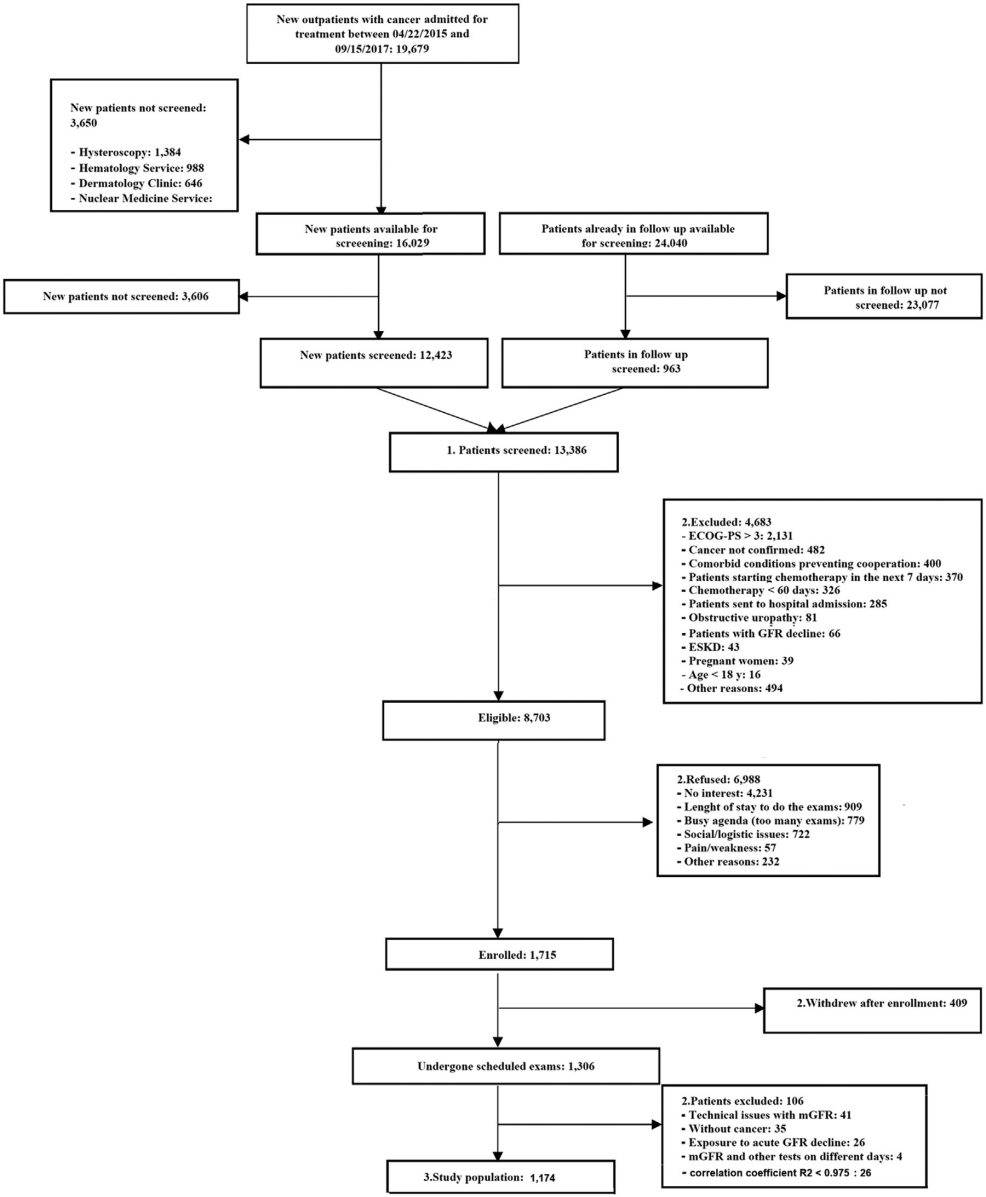
Cohort flow diagram. ECOG, Eastern Cooperative Oncology Group performance status; ESKD, End Stage Kidney Disease. 1, Screened; 2, Not screened; 3, Study population.

**Fig. 2:**
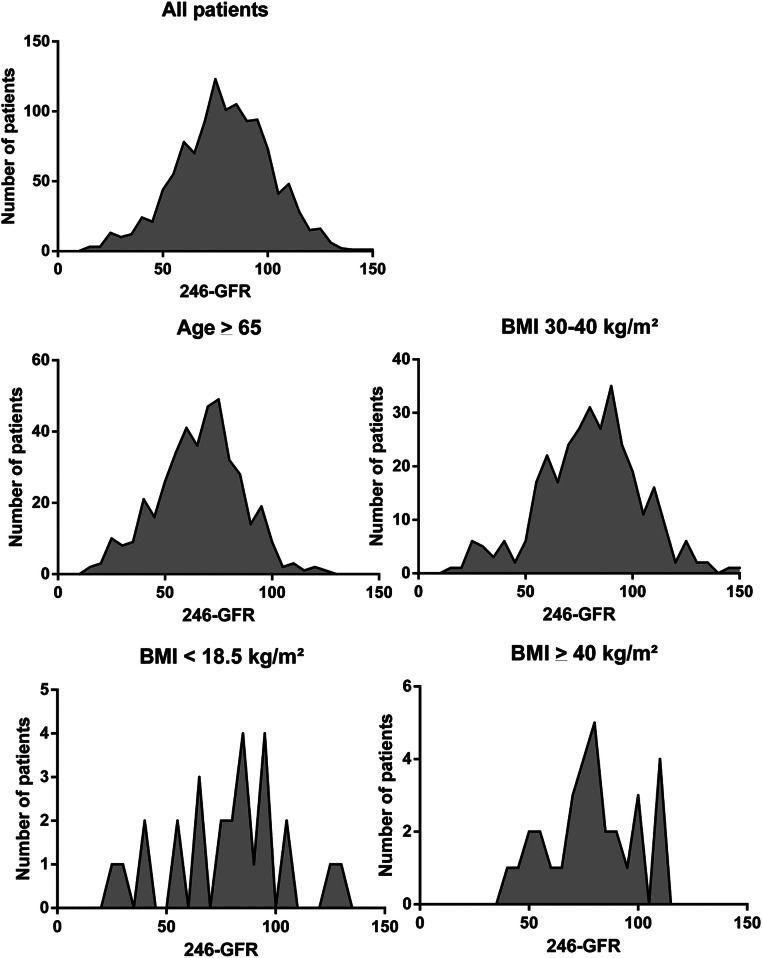
Density plots shows variation among the mGFR for for all patients, age > 65y, BMI < 18.5, 30‒30 or > 40 kg/m^2^.

A total of 13,386 patients were screened between April 22^th^ 2015 and September 15^th^ 2017, from which 1,174 patients agreed to participate and completed the study (Fig. 1). The population consisted of 573 females (48.8%) and 601 males (51.2%), with a mean age of 58.9±13.2 years. There were 413 (35.2%) patients ≥ 65 years. Mean BMI was 27.8±5.4 kg/m², with BMI < 18.5 kg/m^2^ in 26 patients (2.2%), 30‒40 kg/m^2^ in 323 patients (27.5%), and ≥ 40 kg/m^2^ in 32 patients (2.7%). Mean GFR for the 1.174 patients was 79.2 ± 21.9 mL/min/1.73 m^2^, with GFR > 105 mL/min/1.73 m^2^ in 134 patients (11.4%), GFR 90‒105 mL/min/1.73 m^2^ in 242 patients (20.6%), GFR 60‒90 mL/min/1.73 m^2^ in 578 patients (49.2%), GFR 45‒60 mL/min/1.73 m^2^ in 142 patients (12.1%) and GFR < 45 mL/min/1.73 m^2^ in 78 patients (6.6%) (Fig. 2).

Descriptive analysis of the 246-GFR, 24-GFR, and 46-GFR results for the whole population and patients with age ≥ 65 years, GFR, and BMI subgroups are shown in Table 1 (4Gr-GFR results are included in Supplementary Material Table 3).

**Table 1:** GFR means and standard deviations for all population and subgroups: patients ≥ 65 years; BMI < 18.5, 30‒40 or > 40 kg/m²; 246-GFR < 45, 45‒60, 60‒90, 90‒105, > 105 mL/min/1.73 m^2^.

Method	All patients (*n* = 1174)		Age ≥ 65 years (*n* = 413)		BMI < 18.5 (*n* = 26)		BMI 30‒40 (*n* = 323)		BMI > 40 (*n* = 32)	
	mL/min/1.73 m^2^		mL/min/1.73 m^2^		mL/min/1.73 m^2^		mL/min/1.73 m^2^		mL/min/1.73 m^2^	
	Mean	SD	Mean	SD	Mean	SD	Mean	SD	Mean	SD
246-GFR	79.2	21.9	77.7	26.3	77.7	26.3	80.5	23.1	78.4	19.8
24-GFR	79.3	21.6	77.6	26.0	77.6	26.0	80.7	22.7	79.4	19.9
46-GFR	80.8	24.3	78.6	27.4	78.6	27.4	82.6	25.9	78.1	18.6
4Fl-GFR	79.3	19.4	77.1	21.6	77.1	21.6	80.8	20.7	79.8	19.8

Method	GFR > 105 (*n* = 134)		GFR 90‒105 (*n* = 242)		GFR 60‒90 (*n* = 578)		GFR 45‒60 (*n* = 142)		GFR < 45 (*n* = 78)	
	mL/min/1.73 m^2^		mL/min/1.73 m^2^		mL/min/1.73 m^2^		mL/min/1.73 m^2^		mL/min/1.73 m^2^	
	Mean	SD	Mean	SD	Mean	SD	Mean	SD	Mean	SD
246-GFR	115.5	8.3	96.5	4.2	75.7	8.3	53.9	4.0	34.5	8.0
24-GFR	114.9	8.4	96.3	4.5	76.0	8.3	54.7	4.3	35.2	8.5
46-GFR	119.3	16.7	99.4	9.6	77.0	10.3	54.1	4.4	33.9	8.1
4Fl-GFR	110.5	9.1	94.4	4.9	76.5	7.7	56.9	4.2	39.7	8.1

GFR, Glomerular Filtration Rate; BMI, Body Mass Index; n, Number of patients; 246-GFR, GFR based on blood samples, drawn after 2-, 4-, and 6-hours (reference method); 24-GFR, GFR based on blood samples drawn 2- and 4-hours; 46-GFR, GFR based on blood samples, drawn 4- and 6-hours; 4Fl-GFR, GFR based on single sample method proposed by Fleming [16].

**Fig. 3:**
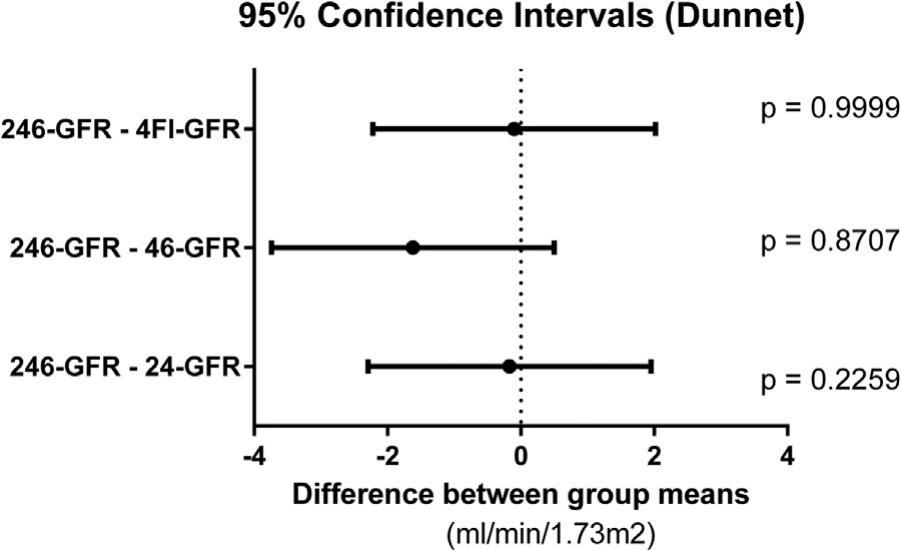
Dunnett's test confidence interval and adjusted p-value for comparison between GFR obtained using slope-intercept with two time-points (24-GFR and 46-GFR) and single-sample at four hours according to Fleming (4Fl-GFR) in relation to the reference method (246-GFR).

ANOVA showed F = 7.041 and p < 0.0001, with a significant difference between 4Gr-GFR and the reference method (246-GFR) by Dunnett's test. The means of 24-GFR, 46-GFR, and 4Fl-GFR had no statistical difference from 246-GFR, as shown in Fig. 3.

Bias, precision, and accuracy (Acc30% and Acc10%) according to the 246-GFR are shown in Table 2, for all patients and subgroups based on BMI, age, and GFR (4Gr-GFRresults are included in Supplementary Material Table 2). Bias was lower than 5 mL/min/1.73 m^2^, except for the SS methods in specific subgroups (4Gr-GFR in patients with BMI ≥ 40 kg/m^2^; both SS methods in patients with GFR > 105 mL/min/1.73 m^2^, 4Fl-GFR in patients with GFR < 45 mL/min/1.73 m^2^). The 46-GFR had lower precision than the other methods considering all patients, mainly due to high GFR and high BMI patients. Acc30% was greater than 98% for all methods in all subgroups, except for both SS methods in patients with GFR < 45 mL/min/1.73 m^2^ (4Gr-GFR = 94.9% and 4Fl-GFR = 89.7%). Acc10% lower than 80% was found for 4Fl-GFR in patients with BMI < 18.5 kg/m^2^ (76.9%), for 46-GFR in patients with 246-GFR between 90‒105 or > 105 mL/min/1.73 m^2^ (74.8% and 58.2%, respectively) and, again, for both SS methods in patients with GFR < 45 mL/min/1.73 m^2^ (4Gr-GFR = 62.8% and 4Fl-GFR = 35.9%).

**Table 2:** Bias, precision and accuracy of 24-GFR,46-GFR, and 4Fl-GFR according to the 246-GFR.

	Method	Bias [95% CI]	Precision	Acc30% (%)	Acc10% (%)
		(mL/min/1.73 m^2^)	(mL/min/1.73 m^2^)		
**All patients (***n* = **1174)**	24-GFR	0.17 [0.06, 0.28]	1.96	100.0%	98.4%
	46-GFR	1.62 [1.21, 2.03]	7.11	99.8%	82.9%
	4Fl-GFR	0.10 [0.00, 0.20]	4.47	99.3%	92.2%
**Age ≥ 65 (***n* = **413)**	24-GFR	0.50 [0.32, 0.68]	1.90	100.0%	96.9%
	46-GFR	0.84 [0.41, 1.26]	4.40	100.0%	93.0%
	4Fl-GFR	1.40 [1.03, 1.77]	3.80	98.8%	88.1%
**BMI < 18.5 (***n* = **26)**	24-GFR	-0.02 [-0.80, 0.76]	1.90	100.0%	100.0%
	46-GFR	0.93 [-0.94, 2.79]	4.61	100.0%	92.3%
	4Fl-GFR	-0.60 [-3.20, 2.00]	6.50	100.0%	76.9%
**BMI 30‒40 (***n* = **323)**	24-GFR	0.20 [-0.04, 0;44]	2.20	100.0%	97.2%
	46-GFR	2.12 [1.27, 2.96]	7.87	99.4%	83.6%
	4Fl-GFR	0.20 [-0.18, 0.58]	4.40	98.5%	92.0%
**BMI ≥ 40 (***n* = **32)**	24-GFR	1.04 [0.36, 1.72]	1.89	100.0%	97.5%
	46-GFR	-0.22 [-2.78, 2.34]	7.10	99.4%	83.9%
	4Fl-GFR	1.45 [-0.62, 3.52]	5.73	98.6%	92.1%
**GFR > 105 (***n* = **134)**	24-GFR	-0.60 [-1.12, -0.08]	3.00	100.0%	99.3%
	46-GFR	3.77 [1.42, 6.12]	13.72	99.3%	58.2%
	4Fl-GFR	-5.00 [-6.17, -3.83]	6.80	100.0%	85.1%
**GFR 90‒105 (***n* = **242)**	24-GFR	-0.20 [-0.40, -0.00]	1.60	100.0%	99.6%
	46-GFR	2.90 [1.83, 3.97]	8.41	99.6%	74.8%
	4Fl-GFR	-2.10 [-2.57, -1.63]	3.70	100.0%	97.5%
**GFR 60‒90 (***n* = **578)**	24-GFR	0.30 [0.17, 0.43]	1.60	100.0%	99.8%
	46-GFR	1.22 [0.81, 1.63]	5.06	100.0%	86.7%
	4Fl-GFR	1.45 [1.23, 1.67]	5.73	98.6%	92.1%
**GFR 45‒60 (***n* = **142)**	24-GFR	0.80 [0.48, 1.12]	1.90	100.0%	98.6%
	46-GFR	0.22 [-0.09, 0.53]	1.88	100.0%	97.9%
	4Fl-GFR	3.10 [2.78, 3.42]	1.90	100.0%	90.8%
**GFR < 45 (***n* = **78)**	24-GFR	0.70 [0.143, 1.26]	2.50	100.0%	82.1%
	46-GFR	-0.57 [-0.84, -0.30]	1.18	100.0%	94.9%
	4Fl-GFR	5.20 [4.35, 6.05]	3.70	89.7%	35.9%

GFR, Glomerular Filtration Rate; BMI, Body Mass Index; n, Number of patients; Acc30%, Percentage of the results lying within 30% of the 246-GFR; Acc10%, Percentage of the results lying within 10% of the 246-GFR; CI, Confidence Interval; 246-GFR, GFR based on blood samples, drawn after 2-, 4-, and 6-hours (reference method); 24-GFR, GFR based on blood samples drawn 2- and 4-hours; 46-GFR, GFR based on blood samples, drawn 4- and 6-hours; 4Fl-GFR, GFR based on single sample method proposed by Fleming [16].

**Fig. 4:**
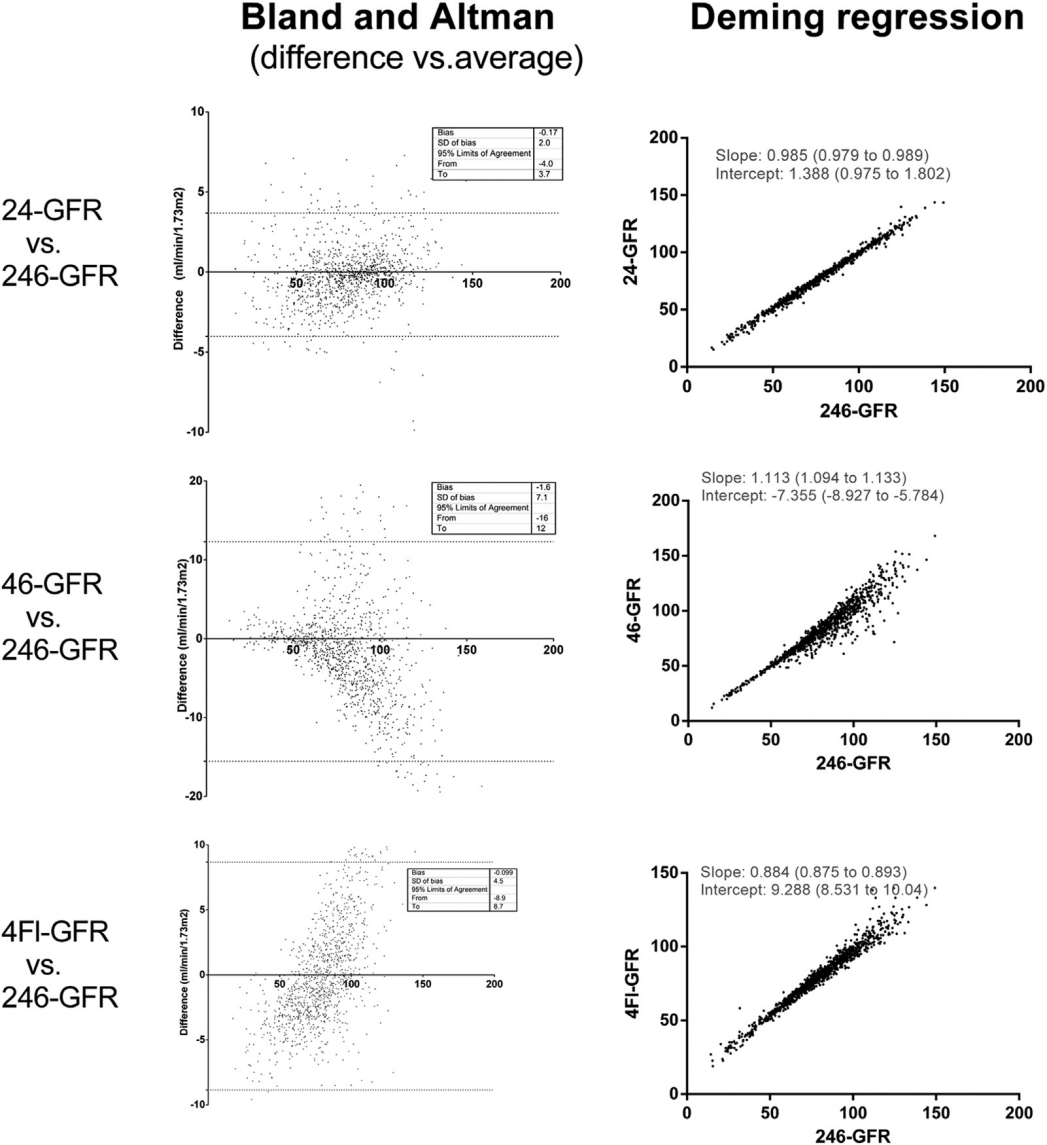
Bland and Altman (B&A) and Deming regression plot of [^51^Cr]CrEDTA GFR evaluated using two time-points (24-GFR and 46-GFR) and single-sample (4Fl-GFR), always in comparison to the reference method (246-GFR). B&A 95% limits of agreement are shown as two dotted lines. Deming regression plot shows the fitted linear model (solid line). Regression parameters were reported as slope/intercept with 95% confidence limits, equations can be expressed as Y = slope*X + intercept.

B&A graphics are shown in Fig. 4. SI 24-GFR shows the lowest dispersion (95% limits of agreement) and bias. For 46-GFR, dispersion increased in patients with GFR > 60 mL/min/1.73 m^2^. SS methods dispersion is also small, although with a detectable bias in 4Gr-GFR (always greater than 246-GFR) and a progressive decrease of 4Fl-GFR in relation to 246-GFR.

Supplementary material presents the results obtained using SI GFR with two samples at two and six-hour (26-GFR); SS GFR according to Groth and Fleming using the two-hour sample (2Gr-GFR and 2Fl-GFR), the six-hour sample (6Gr-GFR and 6Fl-GFR), or the combination of SS results by adjusting sampling times according to the expected GFR (Supplementary Material 2. Results from different combinations of sampling times (SI and SS): Suppl. Table 1: GFR means and standard deviations; Suppl. Table 2: Bias, precision, and accuracy; Suppl. Fig. 1: Bland and Altman analysis).

## Discussion

A large proportion of patients undergoing GFR measurement are cancer patients, accounting for approximately 70% of cases in a UK audit.^29^ but this study is the first, to our knowledge, that compares different SI and SS methods of GFR measures based on EDTA clearance in a cohort composed exclusively of patients with solid tumors. The authors studied a large group of cancer patients in which the three samples showed high fitting to the exponential curve, the same strategy adopted for other comparisons of [^51^Cr]CrEDTA GFR in the literature.^21^^,^^22^ The authors observed a strong correlation between 46-GFR and 246-GFR in all cancer patients, with a similarly high agreement for single-sample measurements in non-obese patients with expected normal renal function. This suggests the potential for adopting simplified procedures in the routine assessment of GFR in oncology.

One-way ANOVA analysis showed no statistically significant difference between 46-GFR and 246-GFR. Low bias, adequate precision, and high accuracy were observed in the analysis of the whole group, and these values were also consistent among extremely obese patients, patients over 65 years, or different GFR ranges (< 45, 45‒60, 60‒90, 90‒105 and > 105 mL/min/1.73 m^2^).

For patients with higher GFR (GFR > 105 mL/min/1.73 m^2^), the earlier double sample, 24-GFR, had better accuracy compared to the later one, 46-GFR. The greater precision of the SI method with early samples is also seen in the B&A analysis. However, in patients with lower GFR the later double sample, 46-GFR performed better than the earlier samples. The authors also compared the results obtained with a single sample with the standard 246-GFR, using two methods (Groth and Fleming). A limitation in this approach to the single-sample plasma clearance method is that time points were pre-defined, and not adjusted to the expected GFR.^21^^,^^22^ The authors tried to address this issue by considering the SS results adjusted to two-, four- or six-hour samples according to the expected GFR (Supplementary Material). SS based on 4-hour samples was analyzed more extensively, as it showed higher accuracy than adjusted or 6-hour sample SS for all subgroups analyzed except GFR < 45.

SS GFR estimated by the Groth method showed a statistically significant difference to the 246-GFR, while the mean GFR obtained using the Fleming method had no statistically significant difference. Both single-sample methods proposed by Growth^13^, ^14^, ^15^ and by Fleming (derived from Jacobsson)^16^^,^^17^ employ mathematical frameworks where the surrogate for extracellular volume, represented by the apparent volume of distribution of the tracer, is determined based on the Body Surface Area (BSA). Additionally, these methods establish a correlation between plasma activity concentration at time (*t*) and the total injected activity, thereby elucidating radiotracer clearance. However, they utilize distinct equations to account for the observed fluctuations in measured GFR (complete equations in supplementary material).

The SS methods were the only ones to present bias greater than 5 mL/min/1.73 m^2^ in specific subgroups (BMI ≥ 40 kg/m^2^; GFR > 105 mL/min/1.73 m^2^; GFR < 45 mL/min/1.73 m^2^). Less than 2% of the patients in all groups presented a difference between the single-sample GFR to the standard larger than 30%, with the exception of patients with GFR < 45 mL/min/1.73 m^2^. Less than 20% of patients presented a difference between the SS GFR to the standard larger than 10%, with the exception of patients with GFR < 45 mL/min/1.73 m^2^ and patients with BMI < 18.5. Less accurate results in the low GFR group were expected, and it is usually recommended not to use SS methods for patients with GFR < 30 mL/min/1.73 m^2^.^30^^,^^31^ In the decision to use SS GFR techniques, it must be also considered that methodological errors may be more difficult to detect.^32^

A limitation of this study is the lack of inulin clearance as the gold standard for measuring GFR. It should be considered that inulin clearance is rarely applied in the clinical setting.^24^ It is noteworthy that the studied group is one of the few with experience in the direct comparison of GFR measured by [^51^Cr]CrEDTA with inulin clearance.^11^ Using multiple combinations of plasma samples collected at two, four, six, and eight hours, [^51^Cr]CrEDTA GFR obtained with two samples at four and six hours presented the best correlation with the inulin clearance in 44 kidney transplant patients (mean inulin clearance = 44.5±17.9 mL/min/1.73 m^2^).

As inulin clearance was not considered viable for a large-scale application in this study, the authors used a group of patients with three samples well fitted to the exponential curve (246-GFR) as a standard to compare the other methods. This choice was made considering the possibility of quality control of the procedure in this group, as a problem in any of the blood collections implies a reduction of the correlation coefficient R^2^.

This study showed a high agreement of 24-GFR and 46-GFR with 246-GFR, and the authors consider that both can be adopted for the evaluation of cancer patients. The preference is for the 46-GFR, not only because of lower complexity but mainly because of the previous study that shows the best agreement with the results of inulin in patients with reduced GFR.^11^ Even considering that the mean GFR in this cohort is higher than that observed in kidney transplant patients, it can be argued that patients with low GFR are the critical ones, who will benefit most from an adjustment in chemotherapy doses. The present study corroborates the low bias and high precision of 46-GFR in patients with 246-GFR < 60 mL/min/1.73 m^2^.

In conclusion, the present study suggests the feasibility of employing [^51^Cr]CrEDTA clearance to evaluate kidney function in patients with solid tumors using different SI and SS methods, with a high agreement between 46-GFR and 246-GFR. The protocol can be tailored according to the clinical characteristics of patients, with the recommendation of delayed samples for patients with expected low GFR. Single-sample methods can also be adopted in specific situations, for non-obese patients with expected normal GFR.

## Conflicts of interest

The authors declare no conflicts of interest.

## Appendix Supplementary materials

Image, application 1
