# Supplementary material for: Comparison of plasma clearance of [51Cr]CrEDTA based on three, two and single samples to measure the glomerular filtration rate in patients with solid tumors: a prospective cross-sectional analysis
Source: Clinics (Sao Paulo). 2024 Jun 29;79:100427. doi: 10.1016/j.clinsp.2024.100427 (PMC11261263; doi:10.1016/j.clinsp.2024.100427)

**CLINICS-D-23-00583_Supplementary Material**

**Supplementary Information** Table of contents.

| **1) Cr-51 EDTA exam** |
| --- |
| a. Preparation |
| b. Administration |
| c. Sample collection and counting |
| d. GFR calculations – two and three plasma samples |
| e. GFR calculations – single plasma samples |
|  |
| **2) Results from different combinations of sampling times (SI and SS)** |
| Suppl. Table 1 GFR means and standard deviations |
| Suppl. Table 2 Bias, precision and accuracy |
| Suppl. Figure 1 Bland and Altman (B&A) analysis |
|  |
| **3) Cr-51 EDTA exam** |
| **a.** Preparation |
| Patients were advised to avoid extenuating exercise the day before the exam.[31] Patients were instructed to fast for 3-hours and received 2-glasses (300 mL) of water 1-hour before Cr-51 EDTA administration. During the 6-hours of the procedure, water and food intake were free, avoiding protein-rich meals. |
| **b.** Administration |
| Two syringes containing about 3.7 MBq of Cr-51 EDTA (IPEN – São Paulo, Brazil) in 1 mL volume were weighted on an analytical balance (Quimis^®^ Q500L210C). One of the syringes was injected into a volumetric flask containing 1000 mL of water and the solution was homogenized 20 times. After 15 minutes, two 2 mL standard samples of the homogenized solution were pipetted. |
| The other syringe´s solution was injected in the patient, followed by 10 mL of saline flush. Both Cr-51 EDTA syringes were weighted after administration of their content in the analytical balance, and the value obtained after subtraction of each syringe's weights before and after administration was considered the Cr-51 EDTA injected mass for both volumetric balloon and the patient. A pancake detector (IEN SPQ-7026^®^/IEN MIR-7026^®^) was used to monitor the patient´s injection site and guarantee that there was no leakage of Cr-51 EDTA. |
| **c.** Sample collection and plasma counting |
| After initial procedures patients stayed in the nuclear medicine facility. Blood samples were collected 2-, 4-, and 6-hours after Cr-51 EDTA injection in a different limb from the site of radiopharmaceutical injection, in tubes containing 0.2 mL of heparin. At each draw, the first 3 mL of blood was discarded and then 10 mL of blood was collected. Subsequently, the blood tubes were centrifuged at 1000g for 10-minutes, and 2 mL of plasma was pipetted from each tube. |
| Samples from volumetric balloon and patient plasma samples were counted for 5-minutes in a well counter using the Cr-51 photopeak of 320 keV as center and with a window of 10%. Background radiation was also counted for 5-minutes and subtracted from each sample counts. Final counts were divided by the volume to obtain results in counts/mL units. |
| **d.** GFR calculations – two and three plasma samples |
| GFR was calculated by the slope-intercept method,[32], using two samples at 2- and 4-hours (24-GFR) or 4- and 6-hours (46-GFR), and three samples at 2-, 4-, and 6-hours (246-GFR). |
| Cr-51 EDTA concentration volume conversion factor (CVCF) was determined multiplying balloon standard samples counts (Cb) by the balloon total volume (Vb) and then multiplying by the weight difference (WD) from the standard syringes before and after Cr-51 EDTA in the balloon as in the following equation: |
| $CVCF=Cb*Vb*Wd$ |
| Patient´s initial distribution volume (DV) of Cr-51 EDTA was determined by dividing the concentration volume conversion factor (CVCF) by the curve intercept (I) in the following equation: |
| $DV=\frac{CVCF}{I}$ |
| Finally, the raw glomerular filtration rate in mL/min was obtained by multiplying the distribution volume (DV) by the curve slope (S): |
| $raw GFR=DV*S$ |
| Raw GFR was corrected for 1.73 m^2^ body surface area (BSA = 0.007184 × Height^0.725^ × Weight^0.425^, according to DuBois equation[26]) and the final GFR was corrected for the early exponential component of the clearance as proposed by Brochner-Mortensen[11] using the following equation: |
| $GFR=\left( 1,0004*BSA corrected GFR \right)-(0,00146*\left( BSA corrected GFR \right)^{2}$ |
| **e.** GFR calculations – single plasma samples |
| Single sample GFR was determined according to the Christensen-Groth technique,[13] using either the 2-hours (2-GFR), 4-hours (4-GFR) or the 6-hours sample (6-GFR), according to the following formula: |
| 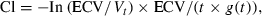 |
| Where: ECV = 8116.6×BSA-28.2 is the extracellular volume, *t* (min) is the time of drawing of the blood sample, BSA (m^2^) the body surface area, and g (*t*) is the function g (*t*)=(0.0000017×*t*-0.00120) × Cl-0.00075×*t*+1.31. |
| Single sample GFR was determined according to the Fleming (derived form Jacobsson) method,[[18]](https://paperpile.com/c/jmG7B5/k8Y9y) using either the 2-hours (2-GFR), 4-hours (4-GFR) or the 6-hours sample (6-GFR), according to the following formula: Cl = t-1 × (-11297 - (4883 × BSA) - (41.9 x t)) + (5862 + (1282 × BSA) + (15.5 × t)) × ln (Vapp(t)) |
| Where: Vapp is the Apparent Volume of Distribution (~ECV), t(min) is the time of drawing of the blood sample, and BSA (m^2^) the body surface area: |
|  |
| **4) Results from different combinations of sampling times (SI and SS)** |
| Results from SI at 2 and 6 hour (26-GFR); SS according to Groth and Fleming methods using the 2-hour (2Gr-GFR and 2Fl-GFR), the 6-hour sample (6Gr-GFR and 6Fl-GFR), or sampling times adjusted to the GFR (SSadj-Gr, SSadj-Fl); additionally, to those included in the manuscript. Sampling times for SSadj were adjusted considering the 2 hours sample for GFR >90 mL/min/1.73 m^2^, the 4h sample for GFR >45–90 mL/min/1.73 m^2^ and the 6 hours sample for GFR <45 mL/min/1.73 m^2^, as an approximation of literature recommendation.[20,21] |

**Supplementary Table 1** GFR means and standard deviations for all population; patients ≥65 years; BMI <18.5, 30‒40 or >40 kg/m²; 246-GFR <45, 45‒60, 60‒90, 90‒105, >105 mL/min/1.73 m^2^.

| **Method** | **All patients (n=1174)** | | **Age ≥65 years (n=413)** | | **BMI <18.5 (n=26)** | | **BMI 30‒40 (n=323)** | | **BMI >40 (n=32)** | |
| --- | --- | --- | --- | --- | --- | --- | --- | --- | --- | --- |
|  | **mL/min/1.73 m^2^** | | **mL/min/1.73 m^2^** | | **mL/min/1.73 m^2^** | | **mL/min/1.73 m^2^** | | **mL/min/1.73 m^2^** | |
|  | **Mean** | **SD** | **Mean** | **SD** | **Mean** | **SD** | **Mean** | **SD** | **Mean** | **SD** |
| 246-GFR | 79.2 | 21.9 | 66.0 | 19.5 | 77.7 | 26.3 | 80.5 | 23.1 | 79.4 | 19.9 |
| 24-GFR | 79.3 | 21.6 | 66.6 | 19.4 | 77.6 | 26.0 | 80.7 | 22.7 | 78.0 | 20.4 |
| 26-GFR | 78.5 | 21.7 | 65.5 | 19.3 | 77.3 | 26.2 | 79.7 | 22.8 | 78.1 | 18.6 |
| 46-GFR | 80.8 | 24.3 | 66.9 | 20.7 | 78.6 | 27.4 | 82.6 | 25.9 | 90.3 | 27.4 |
| 2Gr-GFR | 88.4 | 26.9 | 74.0 | 23.9 | 88.1 | 29.4 | 91.1 | 28.4 | 83.5 | 23.4 |
| 4Gr-GFR | 82.8 | 22.9 | 68.8 | 19.5 | 80.1 | 25.9 | 84.6 | 24.3 | 83.6 | 26.8 |
| 6Gr-GFR | 82.5 | 22.7 | 68.3 | 18.8 | 80.0 | 26.3 | 83.5 | 23.9 | 89.9 | 27.5 |
| SSadj-Gr | 87.6 | 27.2 | 72.1 | 23.7 | 85.8 | 31.4 | 90.2 | 29.1 | 93.1 | 23.8 |
| 2Fl-GFR | 93.9 | 23.7 | 81.3 | 21.5 | 95.9 | 25.7 | 95.1 | 25.0 | 79.8 | 19.8 |
| 4Fl-GFR | 79.3 | 19.4 | 67.5 | 16.9 | 77.1 | 21.6 | 80.8 | 20.7 | 80.9 | 24.4 |
| 6Fl-GFR | 81.6 | 20.9 | 68.4 | 17.6 | 80.7 | 23.9 | 81.9 | 22.1 | 89.5 | 27.1 |
| SSadj-Fl | 89.8 | 27.4 | 74.4 | 24.9 | 90.8 | 31.6 | 91.4 | 28.6 | 78.4 | 19.8 |

| **Method** | **GFR >105 (n=134)** | | **GFR 90‒105 (n=242)** | | **GFR 60‒90 (n=578)** | | **GFR 45‒60 (n=142)** | | **GFR <45 (n=78)** | |
| --- | --- | --- | --- | --- | --- | --- | --- | --- | --- | --- |
|  | **mL/min/1.73 m^2^** | | **mL/min/1.73 m^2^** | | **mL/min/1.73 m^2^** | | **mL/min/1.73 m^2^** | | **mL/min/1.73 m^2^** | |
|  | **Mean** | **SD** | **Mean** | **SD** | **Mean** | **SD** | **Mean** | **SD** | **Mean** | **SD** |
| 246-GFR | 115.5 | 8.3 | 96.5 | 4.2 | 75.7 | 8.3 | 53.9 | 4.0 | 34.5 | 8.0 |
| 24-GFR | 114.9 | 8.4 | 96.3 | 4.5 | 76.0 | 8.3 | 54.7 | 4.3 | 35.2 | 8.5 |
| 26-GFR | 114.4 | 8.6 | 95.6 | 4.6 | 75.1 | 8.2 | 53.5 | 4.1 | 34.3 | 7.9 |
| 46-GFR | 119.3 | 16.7 | 99.4 | 9.6 | 77.0 | 10.3 | 54.1 | 4.4 | 33.9 | 8.1 |
| 2Gr-GFR | 133.3 | 12.7 | 109.2 | 7.9 | 83.4 | 11.5 | 57.0 | 9.6 | 40.4 | 12.1 |
| 4Gr-GFR | 120.6 | 11.7 | 100.5 | 6.0 | 79.2 | 9.0 | 56.5 | 4.7 | 37.4 | 8.6 |
| 6Gr-GFR | 118.3 | 15.7 | 99.4 | 8.6 | 79.5 | 9.3 | 57.4 | 4.9 | 37.2 | 7.9 |
| SSadj-Gr | 132.3 | 9.5 | 112.5 | 6.3 | 90.0 | 10.2 | 66.0 | 8.8 | 49.8 | 12.0 |
| 2Fl-GFR | 110.5 | 9.1 | 94.4 | 4.9 | 76.5 | 7.7 | 56.9 | 4.2 | 39.7 | 8.1 |
| 4Fl-GFR | 113.8 | 13.4 | 97.2 | 7.7 | 79.0 | 8.6 | 58.3 | 4.7 | 38.9 | 8.0 |
| 6Fl-GFR | 115.5 | 8.3 | 96.5 | 4.2 | 75.7 | 8.3 | 53.9 | 4.0 | 34.5 | 8.0 |
| SSadj-Fl | 114.9 | 8.4 | 96.3 | 4.5 | 76.0 | 8.3 | 54.7 | 4.3 | 35.2 | 8.5 |

**Supplementary Table 2** Bias, precision and accuracy of 24-GFR,26-GFR, 46-GFR, 2Gr-GFR, 2Fl-GFR, 4Gr-GFR, 4Fl-GFR, 6Gr-GFR, and 6Fl-GFR according to the 246-GFR.

|  | **Method** | **Bias** | **Precision** | **Acc30% (%)** | **Acc10% (%)** |
| --- | --- | --- | --- | --- | --- |
|  |  | **mL/min/1.73 m^2^** | **mL/min/1.73 m^2^** |  |  |
| **All patients (n=1174)** | 24-GFR | 0.17 | 1.96 | 100.0% | 98.4% |
|  | 26-GFR | -0.68 | 1.84 | 100.0% | 100.0% |
|  | 46-GFR | 1.62 | 7.11 | 99.8% | 82.9% |
|  | 2Gr-GFR | 9.18 | 7.94 | 95.3% | 42.4% |
|  | 4Gr-GFR | 3.60 | 4.30 | 99.5% | 89.9% |
|  | 6Gr-GFR | 3.37 | 7.09 | 98.7% | 81.2% |
|  | SSadj-Gr | 0.19 | 4.19 | 98.7% | 57.6% |
|  | 2Fl-GFR | 14.72 | 5.76 | 88.4% | 7.5% |
|  | 4Fl-GFR | 0.10 | 4.47 | 99.3% | 92.2% |
|  | 6Fl-GFR | 2.40 | 6.63 | 98.9% | 79.6% |
|  | SSadj-Fl | 1.16 | 4.19 | 96.9% | 39.4% |
| **Age ≥65 (n=413)** | 24-GFR | 0.50 | 1.90 | 100.0% | 96.9% |
|  | 26-GFR | -0.56 | 1.35 | 100.0% | 100.0% |
|  | 46-GFR | 0.84 | 4.40 | 100.0% | 93.0% |
|  | 2Gr-GFR | 7.96 | -8.44 | 90.0% | 41.0% |
|  | 4Gr-GFR | 2.75 | 2.95 | 99.0% | 90.8% |
|  | 6Gr-GFR | 2.23 | 4.20 | 99.0% | 86.4% |
|  | SSadj-Gr | 0.40 | 7.10 | 97.8% | 70.9% |
|  | 2Fl-GFR | 15.20 | 6.80 | 77.0% | 5.6% |
|  | 4Fl-GFR | 1.40 | 3.80 | 98.8% | 88.1% |
|  | 6Fl-GFR | 2.40 | 4.50 | 99.0% | 78.9% |
|  | SSadj-Fl | 1.40 | 7.10 | 95.2% | 53.3% |
| **BMI <18.5 (n=26)** | 24-GFR | 0.02 | 1.90 | 100.0% | 100.0% |
|  | 26-GFR | -0.38 | 1.28 | 100.0% | 100.0% |
|  | 46-GFR | 0.93 | 4.61 | 100.0% | 92.3% |
|  | 2Gr-GFR | 10.42 | 7.40 | 92.0% | 42.0% |
|  | 4Gr-GFR | 2.45 | 4.92 | 100.0% | 88.5% |
|  | 6Gr-GFR | 2.31 | 6.57 | 100.0% | 80.8% |
|  | SSadj-Gr | 0.08 | 0.08 | 100.0% | 57.7% |
|  | 2Fl-GFR | 18.20 | 6.00 | 76.9% | 0.0% |
|  | 4Fl-GFR | -0.60 | 6.50 | 100.0% | 76.9% |
|  | 6Fl-GFR | 3.00 | 6.50 | 100.0% | 73.1% |
|  | SSadj-Fl | 1.06 | 0.10 | 96.2% | 15.4% |
| **BMI 30‒40 (n=323)** | 24-GFR | 0.20 | 2.20 | 100.0% | 97.2% |
|  | 26-GFR | -0.87 | 1.92 | 100.0% | 100.0% |
|  | 46-GFR | 2.12 | 7.87 | 99.4% | 83.6% |
|  | 2Gr-GFR | 10.59 | 8.22 | 94.0% | 35.0% |
|  | 4Gr-GFR | 4.13 | 4.34 | 99.7% | 89.8% |
|  | 6Gr-GFR | 3.02 | 7.45 | 99.4% | 80.2% |
|  | SSadj-Gr | 0.10 | 0.10 | 98.8% | 51.7% |
|  | 2Fl-GFR | 14.60 | 5.70 | 88.5% | 8.0% |
|  | 4Fl-GFR | 0.20 | 4.40 | 98.5% | 92.0% |
|  | 6Fl-GFR | 1.40 | 6.90 | 99.4% | 83.6% |
|  | SSadj-Fl | 1.00 | 0.10 | 96.6% | 37.5% |
| **BMI >40 (n=32)** | 24-GFR | 1.04 | 1.89 | 100.0% | 97.5% |
|  | 26-GFR | -0.35 | 1,82 | 100.0% | 100.0% |
|  | 46-GFR | -0.22 | 7.10 | 99.4% | 83.9% |
|  | 2Gr-GFR | 11.92 | 9.27 | 94.0% | 34.0% |
|  | 4Gr-GFR | 5.12 | 7.85 | 99.4% | 89.6% |
|  | 6Gr-GFR | 5.25 | 13.01 | 99.2% | 80.6% |
|  | SSadj-Gr | 0.06 | 0.09 | 98.3% | 51.0% |
|  | 2Fl-GFR | 14.76 | 6.35 | 89.0% | 8.2% |
|  | 4Fl-GFR | 1.45 | 5.73 | 98.6% | 92.1% |
|  | 6Fl-GFR | 2.54 | 10.68 | 99.2% | 83.9% |
|  | SSadj-Fl | 1.03 | 0.10 | 96.6% | 38.0% |
| **GFR >105 (n=134)** | 24-GFR | -0.60 | 3.00 | 100.0% | 99.3% |
|  | 26-GFR | -1.12 | 3.03 | 100.0% | 100.0% |
|  | 46-GFR | 3.77 | 13.72 | 99.3% | 58.2% |
|  | 2Gr-GFR | 17.76 | 7.00 | 99.0% | 15.0% |
|  | 4Gr-GFR | 5.09 | 8.45 | 98.5% | 85.1% |
|  | 6Gr-GFR | 2.81 | 14.37 | 97.0% | 73.1% |
|  | 2Fl-GFR | 16.70 | 4.50 | 99.3% | 11.2% |
|  | 4Fl-GFR | -5.00 | 6.80 | 100.0% | 85.1% |
|  | 6Fl-GFR | - 1.70 | 12.50 | 97.0% | 74.6% |
| **GFR 90‒105 (n=242)** | 24-GFR | - 0.20 | 1.60 | 100.0% | 99.6% |
|  | 26-GFR | -0.91 | 2.15 | 100.0% | 100.0% |
|  | 46-GFR | 2.90 | 8.41 | 99.6% | 74.8% |
|  | 2Gr-GFR | 12.69 | 5.90 | 99.0% | 31.0% |
|  | 4Gr-GFR | 3.95 | 4.43 | 100.0% | 91.7% |
|  | 6Gr-GFR | 2.87 | 7.98 | 98.3% | 85.5% |
|  | 2Fl-GFR | 16.00 | 4.50 | 98.3% | 3.3% |
|  | 4Fl-GFR | -2.10 | 3.70 | 100.0% | 97.5% |
|  | 6Fl-GFR | 0.70 | 7.20 | 99.2% | 89.3% |
| **GFR 60‒90 (n=578)** | 24-GFR | 0.30 | 1.60 | 100.0% | 99.8% |
|  | 26-GFR | -0.60 | 1.58 | 100.0% | 100.0% |
|  | 46-GFR | 1.22 | 5.06 | 100.0% | 86.7% |
|  | 2Gr-GFR | 7.66 | 6.14 | 98.0% | 54.0% |
|  | 4Gr-GFR | 3.44 | 2.95 | 100.0% | 93.3% |
|  | 6Gr-GFR | 3.76 | 5.05 | 99.3% | 81.0% |
|  | 2Fl-GFR | 14.30 | 5.10 | 93.3% | 6.2% |
|  | 4Fl-GFR | 0.80 | 2.70 | 100.0% | 99.7% |
|  | 6Fl-GFR | 3.30 | 4.70 | 99.3% | 84.1% |
| **GFR 45‒60 (n=142)** | 24-GFR | 0.80 | 1.90 | 100.0% | 98.6% |
|  | 26-GFR | -0.43 | 0.80 | 100.0% | 100.0% |
|  | 46-GFR | 0.22 | 1.88 | 100.0% | 97.9% |
|  | 2Gr-GFR | 3.11 | 8.11 | 94.0% | 45.0% |
|  | 4Gr-GFR | 2.60 | 2.17 | 100.0% | 93.0% |
|  | 6Gr-GFR | 3.49 | 2.56 | 100.0% | 83.8% |
|  | 2Fl-GFR | 12.20 | 7.30 | 71.8% | 17.6% |
|  | 4Fl-GFR | 3.10 | 1.90 | 100.0% | 90.8% |
|  | 6Fl-GFR | 4.50 | 2.50 | 100.0% | 72.5% |
| **GFR <45 (n=78)** | 24-GFR | 0.70 | 2.50 | 100.0% | 82.1% |
|  | 26-GFR | -0.17 | 0.44 | 100.0% | 100.0% |
|  | 46-GFR | -0.57 | 1.18 | 100.0% | 94.9% |
|  | 2Gr-GFR | 5.89 | 9.97 | 64.0% | 37.0% |
|  | 4Gr-GFR | 2.88 | 4.06 | 94.9% | 62.8% |
|  | 6Gr-GFR | 2.68 | 1.79 | 96.2% | 78.2% |
|  | 2Fl-GFR | 15.30 | 9.20 | 33.3% | 5.1% |
|  | 4Fl-GFR | 5.20 | 3.70 | 89.7% | 35.9% |
|  | 6Fl-GFR | 4.40 | 2.10 | 96.2% | 37.2% |

GFR, Glomerular Filtration Rate; BMI, Body Mass Index; n, number of patients; Acc30%, Percentage of the results lying within 30% of the 246-GFR; Acc10%, Percentage of the results lying within 10% of the 246-GFR; 246-GFR, GFR based on blood samples, drawn after 2-, 4-, and 6-hours (reference method); 24-GFR, GFR based on blood samples drawn 2- and 4-hours; 26-GFR, GFR based on blood samples drawn 2- and 6-hours; 46-GFR, GFR based on blood samples, drawn 4- and 6-hours; 2Gr-GFR, GFR based on single sample method proposed by Groth at 2-hours; 2Fl-GFR, GFR based on single sample method proposed by Fleming at 2-hours; 4Gr-GFR, GFR based on single sample method proposed by Groth [at](https://paperpile.com/c/aFaAg9/K0McS) 4-hours; 4Fl-GFR, GFR based on single sample method proposed by Fleming at 4-hours; 64Gr-GFR, GFR based on single sample method proposed by Groth [at](https://paperpile.com/c/aFaAg9/K0McS) 6-hours; 6Fl-GFR, GFR based on single sample method proposed by Fleming at 6-hours.

**Supplementary Table 3**. GFR means and standard deviations for all population and subgroups: patients ≥ 65 years; BMI <18.5, 30‒40 or > 40 kg/m²; 246-GFR < 45, 45‒60, 60‒90, 90‒105, > 105 mL/min/1.73 m^2^.

| **Method** | **All patients (n=1174)** | | **Age ≥65 years (n=413)** | | **BMI <18.5 (n=26)** | | **BMI 30‒40 (n=323)** | | **BMI >40 (n=32)** | |
| --- | --- | --- | --- | --- | --- | --- | --- | --- | --- | --- |
|  | **mL/min/1.73 m^2^** | | **mL/min/1.73 m^2^** | | **mL/min/1.73 m^2^** | | **mL/min/1.73 m^2^** | | **mL/min/1.73 m^2^** | |
|  | **Mean** | **SD** | **Mean** | **SD** | **Mean** | **SD** | **Mean** | **SD** | **Mean** | **SD** |
| 246-GFR | 79.2 | 21.9 | 66.0 | 19.5 | 77.7 | 26.3 | 80.5 | 23.1 | 78.4 | 19.8 |
| 24-GFR | 79.3 | 21.6 | 66.6 | 19.4 | 77.6 | 26.0 | 80.7 | 22.7 | 79.4 | 19.9 |
| 46-GFR | 80.8 | 24.3 | 66.9 | 20.7 | 78.6 | 27.4 | 82.6 | 25.9 | 78.1 | 18.6 |
| 4Gr-GFR | 82.8 | 22.9 | 68.8 | 19.5 | 80.1 | 25.9 | 84.6 | 24.3 | 83.5 | 23.4 |

| **Method** | **GFR >105 (n=134)** | | **GFR 90‒105 (n=242)** | | **GFR 60‒90 (n=578)** | | **GFR 45‒60 (n=142)** | | **GFR <45 (n=78)** | |
| --- | --- | --- | --- | --- | --- | --- | --- | --- | --- | --- |
|  | **mL/min/1.73 m^2^** | | **mL/min/1.73 m^2^** | | **mL/min/1.73 m^2^** | | **mL/min/1.73 m^2^** | | **mL/min/1.73 m^2^** | |
|  | **Mean** | **SD** | **Mean** | **SD** | **Mean** | **SD** | **Mean** | **SD** | **Mean** | **SD** |
| 246-GFR | 115.5 | 8.3 | 96.5 | 4.2 | 75.7 | 8.3 | 53.9 | 4.0 | 34.5 | 8.0 |
| 24-GFR | 114.9 | 8.4 | 96.3 | 4.5 | 76.0 | 8.3 | 54.7 | 4.3 | 35.2 | 8.5 |
| 46-GFR | 119.3 | 16.7 | 99.4 | 9.6 | 77.0 | 10.3 | 54.1 | 4.4 | 33.9 | 8.1 |
| 4Fl-GFR | 120.6 | 11.7 | 100.5 | 6.0 | 79.2 | 9.0 | 56.5 | 4.7 | 37.4 | 8.6 |

GFR, Glomerular Filtration Rate; BMI, Body Mass Index; n, number of patients; 246-GFR, GFR based on blood samples, drawn after 2-, 4-, and 6-hours (reference method); 24-GFR, GFR based on blood samples drawn 2- and 4-hours; 46-GFR, GFR based on blood samples, drawn 4- and 6-hours; 4Gr-GFR, GFR based on single sample method proposed by Growth;[13] 4Fl-GFR, GFR based on single sample method proposed by Fleming.[16]

**Supplementary Figure 1** Bland and Altman (B&A) analysis of Cr-51 EDTA GFR evaluated using two time-points (24-GFR, 26-GFR, and 46-GFR) and single-sample (2Gr-GFR, 2Fl-GFR, 4Gr-GFR, 4Fl-GFR, 6Gr-GFR, 6Fl-GFR, SSAdjusted_Groth, and SSAdjusted_Fleming), always in comparison to the reference method (246-GFR). 95% limits of agreement are shown as two dotted lines.


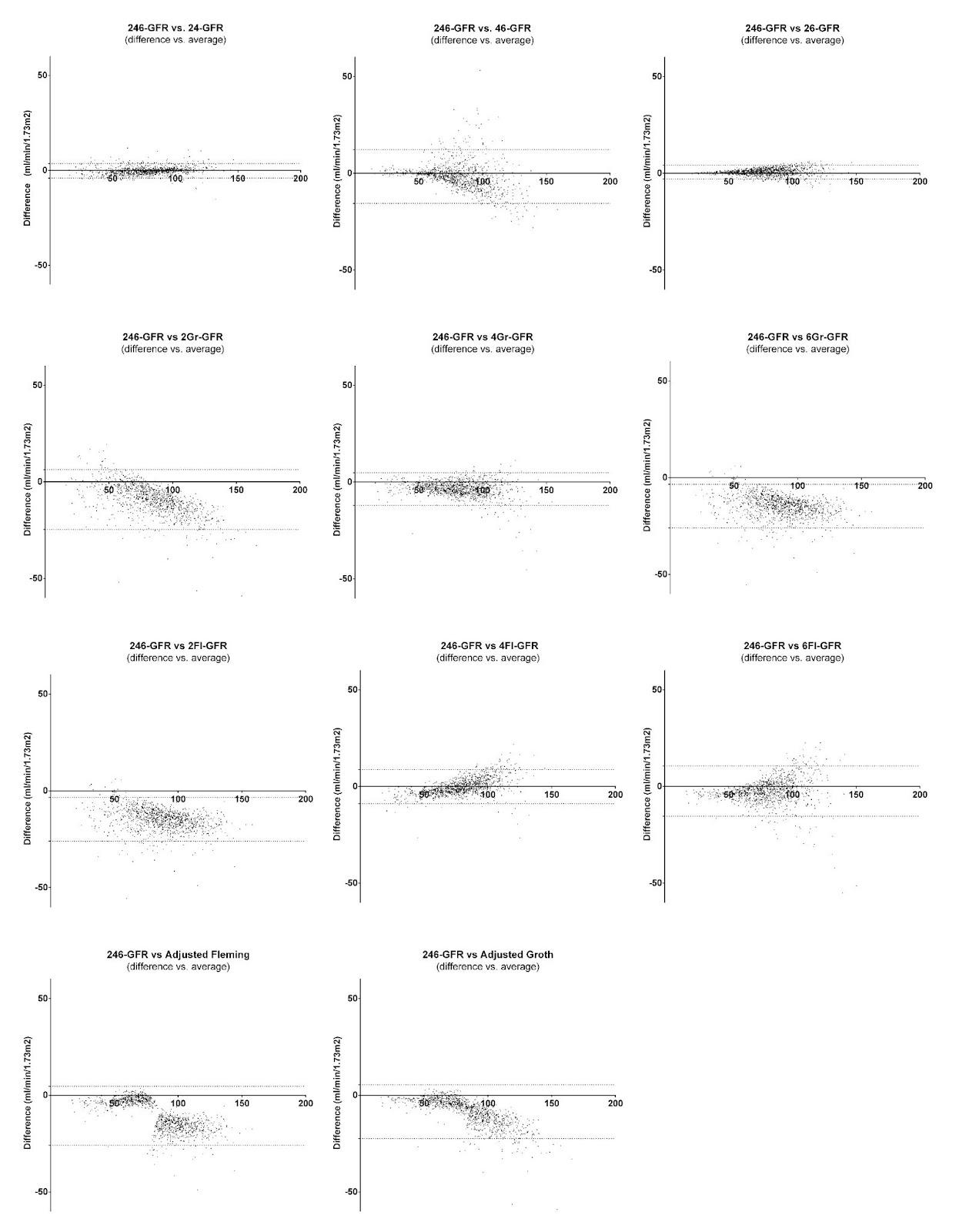

Supplement: Supplementary file 1 [file mmc1.docx]
